# Real-World Experience of mHealth Implementation in Clinical Practice (the Box): Design and Usability Study

**DOI:** 10.2196/26072

**Published:** 2021-12-16

**Authors:** Tom Biersteker, Alexander Hilt, Enno van der Velde, Martin Jan Schalij, Roderick Willem Treskes

**Affiliations:** 1 Department of Cardiology Leiden University Medical Center Leiden Netherlands

**Keywords:** eHealth, mHealth, remote patient monitoring, cardiology, patient satisfaction, patient empowerment, mobile phone

## Abstract

**Background:**

Mobile health (mHealth) is an emerging field of scientific interest worldwide. Potential benefits include increased patient engagement, improved clinical outcomes, and reduced health care costs. However, mHealth is often studied in projects or trials, and structural implantation in clinical practice is less common.

**Objective:**

The purpose of this paper is to outline the design of *the Box* and its implementation and use in an outpatient clinic setting. The impact on logistical outcomes and patient and provider satisfaction is discussed.

**Methods:**

In 2016, an mHealth care track including smartphone-compatible devices, named *the Box*, was implemented in the cardiology department of a tertiary medical center in the Netherlands. Patients with myocardial infarction, rhythm disorders, cardiac surgery, heart failure, and congenital heart disease received devices to measure daily weight, blood pressure, heart rate, temperature, and oxygen saturation. In addition, professional and patient user comments on the experience with the care track were obtained via structured interviews.

**Results:**

From 2016 to April 2020, a total of 1140 patients were connected to the mHealth care track. On average, a Box cost €350 (US $375), not including extra staff costs. The median patient age was 60.8 (IQR 52.9-69.3) years, and 73.59% (839/1140) were male. A median of 260 (IQR 105-641) measurements was taken on a median of 189 (IQR 98-372) days. Patients praised the ease of use of the devices and felt more involved with their illness and care. Professionals reported more productive outpatient consultations as well as improved insight into health parameters such as blood pressure and weight. A feedback loop from the hospital to patient to focus on measurements was commented as an important improvement by both patients and professionals.

**Conclusions:**

In this study, the design and implementation of an mHealth care track for outpatient follow-up of patients with various cardiovascular diseases is described. Data from these 4 years indicate that mHealth is feasible to incorporate in outpatient management and is generally well-accepted by patients and providers. Limitations include the need for manual measurement data checks and the risk of data overload. Moreover, the tertiary care setting in which *the Box* was introduced may limit the external validity of logistical and financial end points to other medical centers. More evidence is needed to show the effects of mHealth on clinical outcomes and on cost-effectiveness.

## Introduction

### Background

The World Health Organization defines mobile health (mHealth) as “a component of electronic health (eHealth), which involves the use of a mobile phone, patient monitoring devices, and other wireless devices to support medical and public health practise” [1]. It is a growing industry and field of research interest, with over 300,000 health apps now being available in major app stores and 1697 hits on PubMed being available in 2019 versus 319 in 2013 [2].

In 2019, 97% of all Dutch inhabitants had access to broadband internet, and 84% used a smartphone to browse the internet [3,4]. This is consistent with other Western countries [4,5]. With most of the Western population using smartphones and health care models becoming increasingly patient-centered, there is a promise for mHealth to change the future of health care [6]. Although sometimes described as a hype with scarce concise scientific projects or evidence [7], mHealth presents opportunities to increase patient engagement, improve clinical outcomes, and reduce health care costs [8,9]. In cardiovascular outpatient care, health care providers and patients are positive toward the potential that mHealth holds [10-12].

In 2016, mHealth was introduced in outpatient care in the department of cardiology at a large tertiary medical center in the Netherlands. This project, named *the Box*, equipped patients with mHealth devices that were handed out at discharge and came in a box for easier transportation. It has been the main focus to make this type of care accessible to every patient with a low threshold for participation [13].

### Objectives

The purpose of this paper is to outline the design of *the Box* and its implementation and use in the outpatient clinic setting. It presents the results of 4 years of structural implementation; logistical and clinical processes as well as reported patient and physician satisfaction are discussed.

## Methods

### Project Design and Evolvement

The Leiden University Medical Center (LUMC) delivers tertiary care for cardiovascular patients, such as primary percutaneous coronary interventions and advanced cardiac surgery as well as atrial and ventricular ablation procedures. Outpatient care of specific patient populations, such as patients who had a myocardial infarction (MI), patients who underwent pulmonary vein isolation for atrial fibrillation (AF), patients with a diagnosis of advanced heart failure (HF), and patients after implantable cardioverter-defibrillator implantation, has been standardized into care tracks. Patients were seen at the outpatient clinic by a nurse practitioner (NP) who was supervised by a consultant cardiologist. This has been described in detail in a previous study [14]. In 2015, it was hypothesized that some of these protocols could be executed via mHealth, as follows:

1. Replacing physical outpatient clinic visits by digital visits via the webcam, as this was hypothesized to be more patient-friendly by saving the patient time and money.
2. Introducing patient home monitoring. As such, patients could review measurements such as blood pressure or heart rate, involving them more in the treatment of their condition. It was hypothesized that by increasing the number of data points, abnormal trends such as high blood pressure could be detected earlier.

As such, an mHealth initiative called *the Box* was launched. For this initiative, smartphone-connectible, consumer-grade health monitoring devices were used, and outpatient contact moments were replaced with video consultations. *The Box* was started at the cardiology department of the LUMC in April 2016, at first as a part of a randomized controlled trial (RCT), registered at ClinicalTrials.gov (NCT02976376). The methods and results of this specific RCT have been described previously [13,15]. In 2018, it was decided to start another study on patients who underwent cardiac surgery, which hypothesized an increased detection of postoperative AF with the use of mHealth devices as well as increased patient satisfaction and empowerment [16]. This study began recruitment in November 2018 and is still ongoing. It has been registered at ClinicalTrials.gov (NCT03690492).

**Figure 1:**
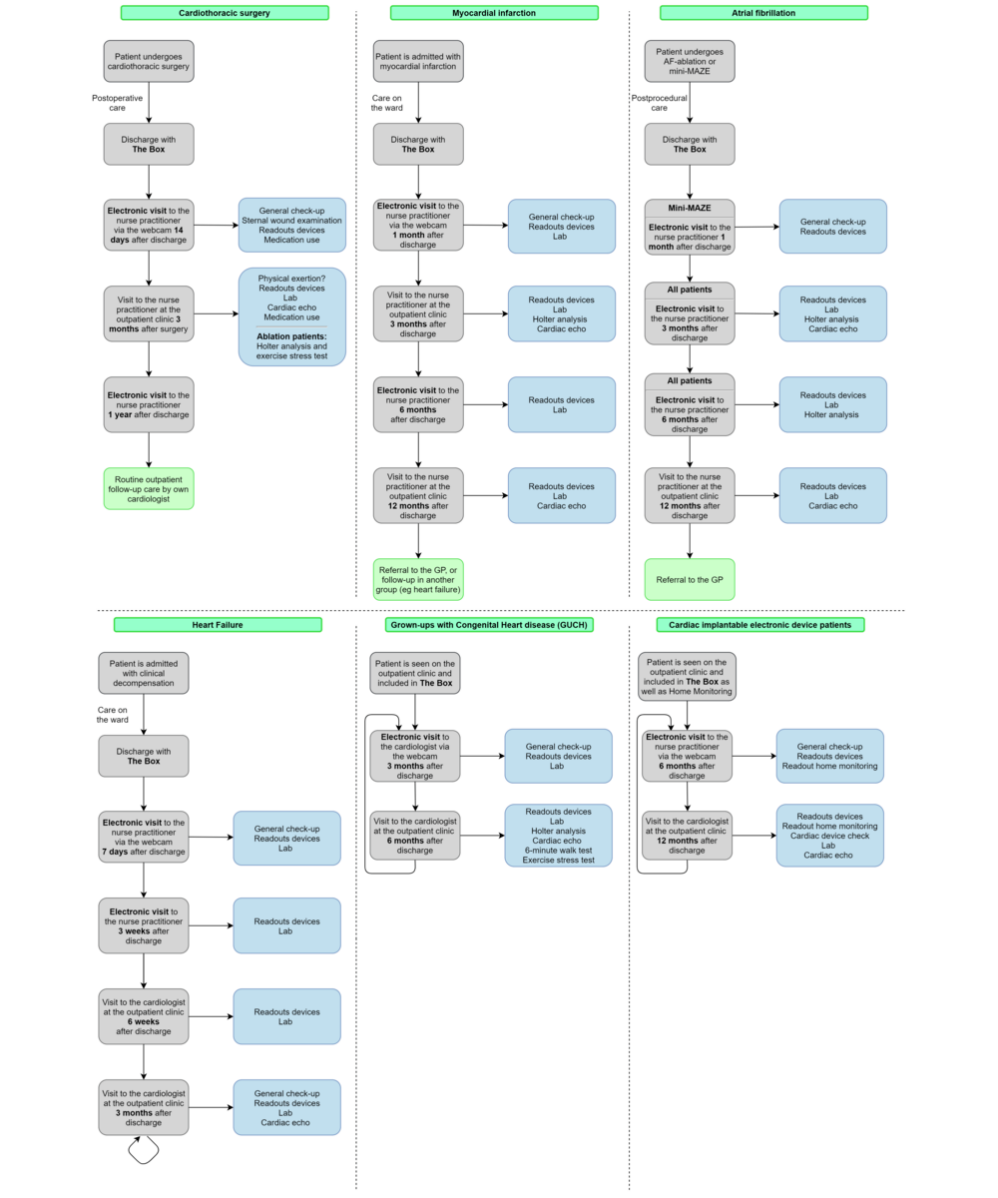
Protocols for follow-up with *the Box*. AF: atrial fibrillation; GP: general practitioner.

Simultaneously, positive first results regarding the RCT led to the introduction of *the Box* as standard care to additional patient groups other than those who had an MI or underwent cardiac surgery; *the Box* was introduced to patients who underwent catheter pulmonary vein isolation and patients with HF, to those after implantation of an implantable cardiac defibrillator or cardiac resynchronization therapy device for any reason, and to grown-ups with congenital heart (GUCH) disease. All currently used outpatient follow-up protocols are listed in Figure 1. Follow-up of the patients was primarily the responsibility of the NPs who handled one patient group each. Measurement results were checked by NPs, and video consultations were also carried out. The video consultations are with regard to discussion topics (symptoms, side effects of medication, etc) comparable with physical outpatient clinic visits.

### Contents of the Box

**Figure 2:**
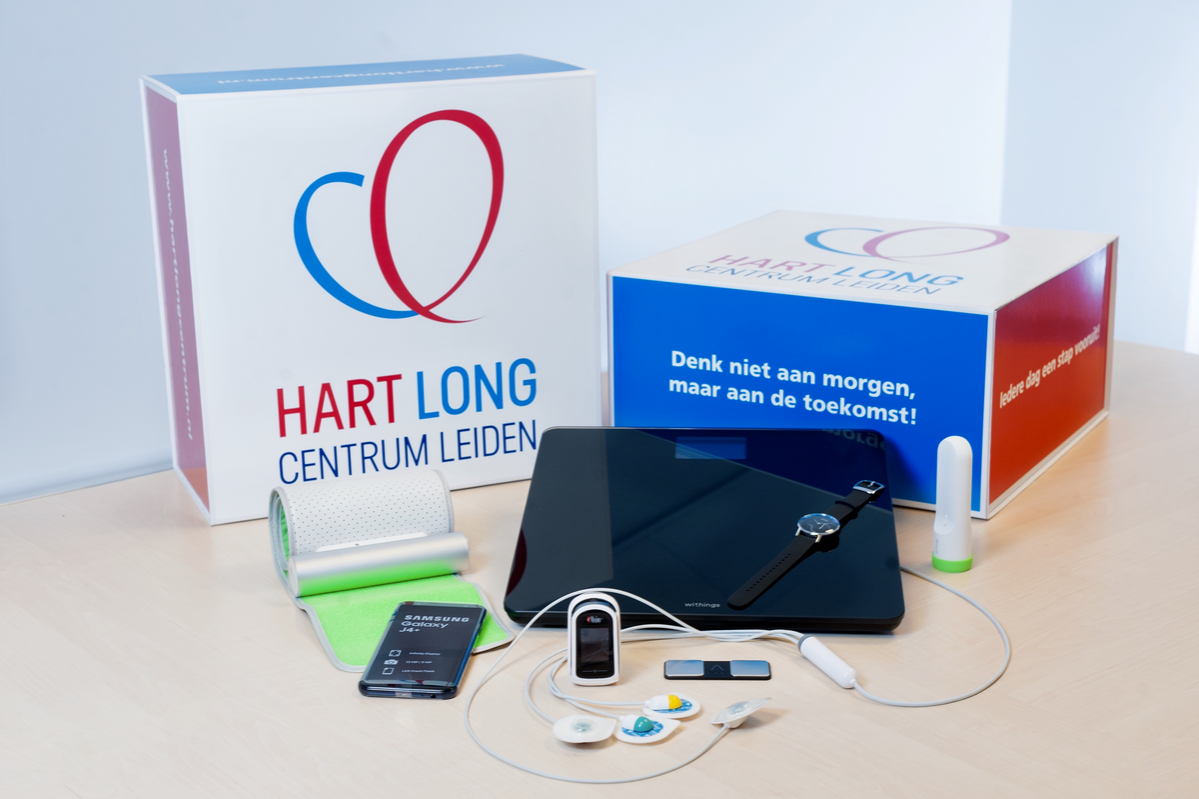
*The Box* with all mobile health devices.

Figure 2 shows a Box with all mHealth devices that are currently being used, as described in an earlier study [16]. Patients received mHealth devices depending on their specific disease. Table 1 summarizes the requested measurement frequency, intended follow-up duration, and number of devices per Box type.

**Table 1:** The measuring frequency and devices provided per patient group (N=1140).

Devices	Measuring frequency and follow-up duration					
	Myocardial infarction (n=449); thrice per week; 12-month follow-up	Cardiac surgery (n=290); thrice per week^a^; 3-month follow-up	Atrial fibrillation (n=260); once per week; 12-month follow-up	Device patients (n=71); thrice per week; follow-up differs per user	Heart failure (n=65); thrice per week; indefinite follow-up	Grown-ups with congenital heart (n=29); twice per week; indefinite follow-up
Blood pressure monitor	✓^b^	✓	✓	✓	✓	✓
Weight scale	✓	✓	✓	✓	✓	✓
Pedometer	✓	✓	✓	✓	✓	✓
Thermometer		✓				
AliveCor Kardia	✓	✓		✓	✓	✓
CardioSecur^c^		✓	✓			
Pulse oximeter		✓				

^a^Daily measurements during the first 2 weeks after discharge.

^b^Device used.

^c^CardioSecur measurements: once every week, plus an extra registration when there are complaints of palpitations, or when the AliveCor Kardia detects possible atrial fibrillation.

A blood pressure monitor, weight scale, thermometer, and activity tracker (pedometer) were provided by Withings. Since 2019, multiple versions of the Withings pedometer, a wristwatch, have been used; some versions (Withings Move ECG and Scanwatch) allow lead-I electrocardiogram (ECG) devices to be made. *The Box* could furthermore contain a pulse oximeter by Masimo, an AliveCor Kardia single lead ECG device (AliveCor Inc) or a CardioSecur, which is a 4-electrode EASI-derived ECG device (Personal MedSystems GmbH). These devices are all consumer-grade, Conformité Européenne–marked, and available in the public market. All devices are smartphone-connectible via Bluetooth or through a wire in the case of CardioSecur and managed in the device-dedicated smartphone apps. Apart from the CardioSecur ECG device and pulse oximeter, all devices were gifted to the patient. On average, one Box with its contents costs the cardiology department of the LUMC a total amount of €350 (US $375), not including extra staff costs. *The Box* is not sponsored by the manufacturers of the devices.

### Installation Process and Support

Patients individually received installation instructions from technical assistants who had no medical background but received specific training. All relevant apps were installed, and all devices were connected to the patient’s smartphone via Bluetooth upon discharge. If a patient did not own a smartphone, a loan device was provided, which was returned after the patient’s follow-up was complete. Furthermore, patients received ample instructions on device operation as well as detailed manuals on the use of all individual devices and video consultation. Moreover, technical assistants ran a helpdesk which could be called by patients in case of technical issues with the devices of *the Box*. Patients were visited at home whenever the technical assistants were unable to resolve a technical issue by telephone.

### Connectibility

The data from the Withings devices were connected to the patient’s electronic medical record (EMR) via the Withings application programming interface via a specific authorization protocol (OAUTH2). CardioSecur ECG registrations were saved to the servers of the manufacturer located in Germany and could be checked by the NP on a web-based dashboard. The patient emailed the single lead ECG registrations and pulse oximeter data to the LUMC. When a rhythm disturbance was diagnosed by the NP, the ECG was manually added to the EMR.

### Measurements and Feedback

Patients received automated feedback from the manufacturer’s apps based on the readouts of the devices. Measurement results were checked by NPs 2 to 3 times per week after passing through an algorithm. This algorithm, programmed by software developers of the cardiology department of the LUMC, flagged abnormal results if the measurements exceeded a certain limit. As such, the upper and lower limits of measurement results, such as blood pressure and weight, could be set per patient individually. The limits were determined at the start of the use period of *the Box* by the responsible NP.

Manual feedback by the NP was provided only in the case of anomalies. As all used devices are consumer grade rather than medical grade, therefore lacking scientifically proven accuracy, this feedback was based on trends rather than individual measurements. Patients were instructed of this *no news is good news* method as well as NPs looking at trends, which was also clearly stated in the provided manuals. However, patients could contact the NP with their measurement results when they felt uncomfortable. Most importantly, though, all patients were instructed to use *the Box* in the outpatient setting but not to use it in case of emergencies. This was communicated during face-to-face instructions by the technical assistants and in all manuals provided with *the Box*.

### Video Consultation

As shown in Figure 1, several protocolled outpatient clinic visits were replaced by video consultations. The patient communicated with their NP via a secured webcam (Webcamconsult) connection. The contents of video consultations and in-office outpatient clinic visits were comparable.

### Patient Privacy

To use mHealth devices, patients must register for the smartphone app. This app is developed and owned by the device manufacturer. As data safety and patient privacy are a big concern in eHealth [17], this raises privacy concerns as patient data are stored on the manufacturer’s servers. To protect patient privacy, patients were provided with an email address containing no personal or any other relatable information. The domain of these email addresses was owned by the LUMC. The account details were exclusively known to the patient and the LUMC, the passwords were randomly generated, and, in every case, the date of birth was January 1, 1950. With this alias, the patient did not have to share personal information such as their name, gender, or date of birth with the manufacturers of the mHealth devices. Moreover, device manufacturers could not contact the patients directly. Importantly, because of working with anonymized accounts, no patient information could be obtained by a third party in case of a data breach concerning the mHealth device accounts.

At the end of the use period of *the Box*, the randomly generated accounts were disconnected from the EMR. This was also discussed with patients at the start of their Box period. This prevented patients from indefinitely sending in their data when their care may have been transferred to another institution or a general practitioner.

### Outcome Measure: Patient and Professional Experiences With the Box

#### Patient Satisfaction

To understand and measure patient satisfaction on the usability and experience of *the Box*, 14 qualitative interviews have been conducted over the course of 2017 until 2019. For these interviews, 14 patients were randomly selected from the RCT. Moreover, to understand patient satisfaction in patients with a low socioeconomic status (SES), 10 in-depth interviews were conducted, mainly focusing on the provision of information and communication by the care team. These patients were selected from the Box 2.0 study.

All qualitative data could be summarized into 5 themes regarding the use of *the Box*, namely, general instructions and information provision, the distribution of *the Box* by the hospital, taking home measurements, the video consultation, and finally the quality of provided support. Patients with a low SES completed additional questions on information provision and communication.

#### Professional Experience

Finally, the NPs and their supervisors were asked to share their experiences, thoughts, and comments. This team has worked with *the Box* and its patients daily since 2016, checking the measurement results and conducting video consultations.

### Data Analysis

Content analysis was used for all qualitative data to structure the output provided by patients and professionals. Authors TEB and ADH structured outcomes into different themes related to the process of using *the Box*.

## Results

### Demographics of All Box Patients

Patient demographics are shown in Table 2. From April 2016 to April 2020, a total of 1164 boxes were handed out to 1140 patients. A total of 24 patients were included in 2 protocols. Of the 24 patients, 20 (83%) used *the Box* after an MI, who were then switched to a postsurgery Box after they underwent coronary artery bypass grafting. The other 17% (4/24) of patients with 2 boxes used a Box after cardiac surgery, after which they were diagnosed with AF, who eventually needed to be treated with pulmonary vein isolation. As such, they used *the Box* for follow-up after AF ablation.

**Table 2:** Group characteristics of Box patients (N=1140).

Group characteristics	Total (n=1140)	Myocardial infarction (n=449)	Cardiac surgery (n=290)	Atrial fibrillation (n=260)	Device patients (n=71)	Heart failure (n=65)	Grown-ups with congenital heart (n=29)
Sex (male), n (%)	839 (73.6)	336 (75.5)	221 (76.2)	185 (71.2)	55 (77.5)	40 (60.1)	16 (55.2)
Age (years), median (IQR; range)	60.8 (52.9-69.3; 21.2-83.0)	59.9 (52.0-67.8; 32.7-83.0)	61.2 (53.9-69.5; 21.6-80.9)	62.0 (56.9-69.5; 34.8-78.9)	66.3 (59.7-72.6; 44.3-79.1)	67.4 (52.2-72.9; 32.8-80.0)	46.4 (43.9-49.6; 21.2-57.7)
Number of measurements, median (IQR; range)	260 (105-641; 1-3159)	336 (133-790; 2-3159)	295 (159-504; 2-2537)	54 (16-128; 2-993)	867 (503-1177; 1-2010)	337 (145-492; 6-1882)	169 (62-368; 11-1675)
Number of days of measurements taken, median (IQR; range)	189 (98-372; 1-1216)	296 (120-466; 1-1216)	165 (87-338; 1-846)	137 (43-232; 1-519)	303 (230-376; 1-468)	142 (99-177; 3-246)	144 (104-179; 2-230)
Travel distance (kilometers), median (IQR; range)	14.8 (7.0-24.3; 1.0-2075.0)	10.5 (5.1-21.2; 1.0-571.0)	14.8 (7.0-23.0; 1.0-2075.0)	16.6 (10.1-28.7; 1.0-193.6)	17.3 (7.0-28.0; 3.1-121.1)	14.3 (7.0-20.9; 2.6-105.8)	50.1 (22.9-119.6; 5.1-230.9)

**Figure 3:**
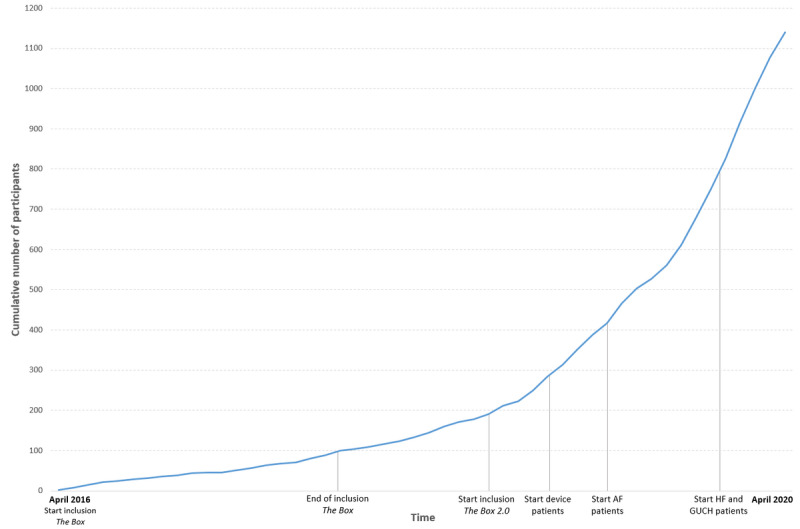
The cumulative number of Box patients over time. *The Box*: randomized controlled trial randomizing 200 patients after myocardial infarction to either the Box or regular follow-up. *The Box 2.0*: trial including 350 post–cardiac surgery patients comparing them with 350 historic control patients. Device patients: patients with an implantable cardioverter-defibrillator or cardiac resynchronization therapy device (patients with AF: patients who underwent atrial fibrillation ablation). AF: atrial fibrillation; GUCH: grown-ups with congenital heart; HF: heart failure.

The median age of all patients of *the Box* was 60.8 (IQR 52.9-69.3) years, with GUCH disease being the youngest (median 46.4, IQR 43.9-69.6 years) and patients with HF being the oldest (median 67.4, IQR 52.2-72.9 years). In total, 73.59% (839/1140) of patients were male. The median number of measurements taken was 260 (IQR 105-641). There was a large between-group variation, which could be explained by the difference in the number of devices used and the difference in follow-up time. The median number of days on which patients conducted at least one measurement was 189 (IQR 98-372). This might be explained by the difference when each group has started using *the Box*, as shown in Figure 3.

**Figure 4:**
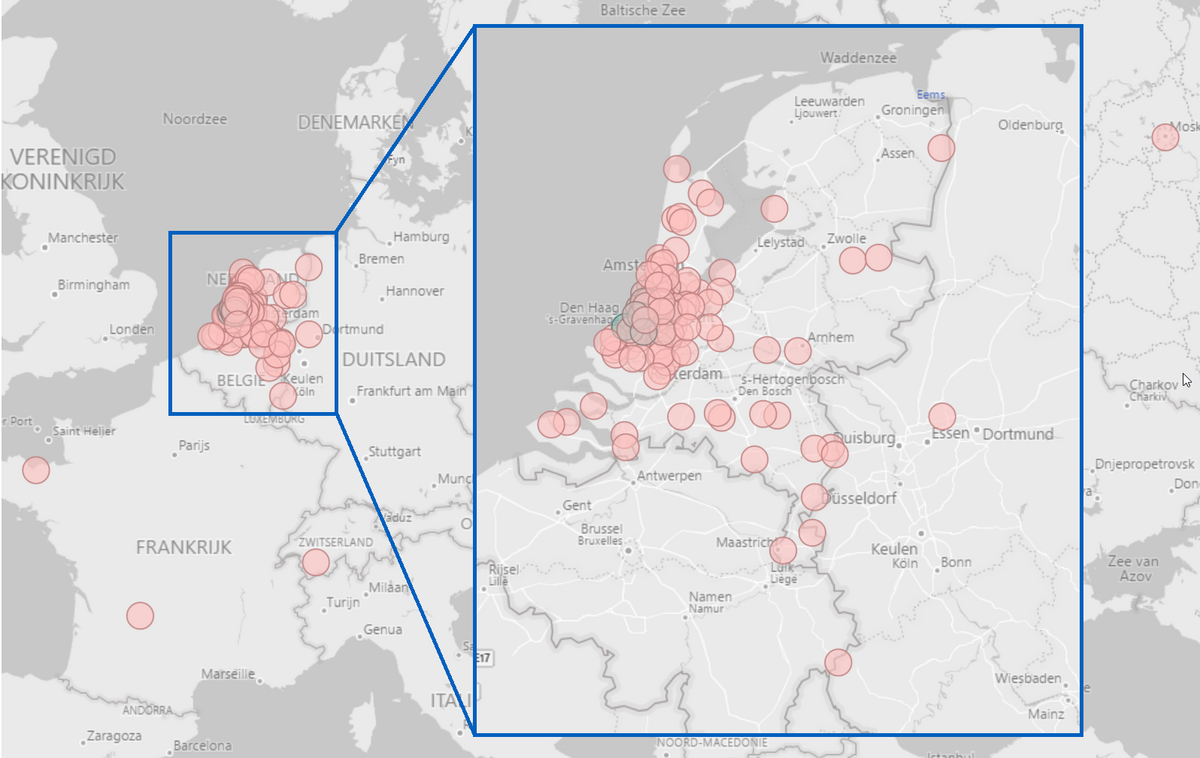
Patient spread throughout the Netherlands and Europe. The red dots represent patient locations, and the green dot represents the location of the Leiden University Medical Center.

The mean travel distance to the hospital was 14.8 (IQR 7.0-24.3) km. Most Box patients live relatively close to the LUMC. Some, however, live outside the Netherlands, with the furthest patients living in Thailand. Figure 4 shows the locations of all 1140 Box patients throughout the Netherlands and Europe.

A total of 19,450 single lead Kardia ECGs were sent in (patient’s mean 39; IQR 21-67) by 449 patients. The number of CardioSecur ECGs was 2125 (patient’s mean 8; IQR 6-13) in 290 cardiac surgery patients. The AF cohort of 260 patients made 2910 CardioSecur ECGs (patient’s mean 11; IQR 7-17). The large difference between the MI group and the other 2 groups regarding ECG measurements is because the follow-up of the MI population is longer than any other group and because they were requested to send in a registration 3 times per week. The other groups only did so once every week but also when the Kardia indicated a possible rhythm disturbance and when patients experienced palpitations.

### Box Distribution

*The Box* started with 200 patients who were included in the RCT in a 1:1, randomized fashion between receiving mHealth follow-up with a Box or regular outpatient follow-up. In November 2017, the last patient was included in this RCT, and as such, 100 boxes were distributed. Thereafter, *the Box* was continued as a standard of care for patients who had MI. In November 2018, *the Box 2.0* started including patients who underwent cardiac surgery. In February 2019, *the Box* became the standard outpatient care for patients with a cardiac device, and in May 2019, *the Box* became the standard outpatient care for patients after AF ablation. Finally, from November 2019 onward, the GUCH disease and patients with HF received *the Box*. Figure 3 shows the cumulative number of patients over time. From 2016 to 2019, 44, 65, 175, and 542 boxes were distributed annually. In 2020, a distribution of 1100 boxes was expected.

### Patient Interviews

#### Overview

The 24 patients who were interviewed had a median age of 61.3 (IQR 48.4-69.7) years. In total, 14 of them participated in *the Box* in 2017, 7 in 2018, and 3 in 2019. Generally, the patients stated *the Box* to be a useful tool for longer use than the intended period of 1 year in patients with MI. Most (21/24, 88%) patients felt *looked after* with the use of *the Box*; however, 33% (8/24) of patients would have preferred an improved feedback system. Textboxes 1 and 2 show the most often heard positive and negative statements regarding the use of *the Box*.

**Most frequent positive comments.**

**Positive comments about *the Box***

- “*The Box* is easy to use.”
- “*The Box* stimulates me to go out: my physical condition has improved and I feel less tired.”
- “I know more about my illness now that I’m learning normal blood pressure values and ECG readings from taking daily measurements.”
- “It is reassuring to know that professionals look after me, also in my own environment.”
- “I feel more confident in my body since the use of the mHealth devices.”
- “I, but also my family, are more aware of potential lifestyle improvements.”
- “I better understand now that patients have a responsibility in their rehabilitation, and *The Box* is a tool that helps me do so.”

**Most frequent negative comments.**

**Negative comments about *the Box***

- “I would have preferred a feedback system, which reassures patients that the measurements are looked at.”
- “*The Box* confronts me with my illness on a daily basis.”
- “*The Box* is too big.”
- “It does not yet feel like the Box connects seamlessly with other rehabilitation programs.”

#### General Instructions and Provision of Information

Most patients (22/24, 92%) reported the installation process to be successful before they left the hospital, although at the same time, an equally large group of patients (22/24, 92%) recalled that the amount of information was too much to remember. In total, 21% (5/24) of patients had questions within a day after *the Box* was installed, of whom, 60% (3/5) were able to solve their questions with the manuals of *the Box*. However, no patients stated that *the Box* was too complicated for them to be able to start with.

#### Distribution Phase

All the patients valued the personal instructions, and no remarks were made on how this process took place. Out of the 24 patients, 8 (33%) patients would have preferred to install *the Box* on their own. A total of 79% (19/24) of patients were satisfied with receiving instructions in the hospital, and 21% (4/24) of patients preferred it to take place after discharge from the hospital.

#### Home Measurements

Patients took a median of 10 (IQR 5-15) minutes daily to take their measurements. A total of 71% (17/24) of patients trusted the validity of the measurements, whereas 4% (1/24) did not. The other 25% (6/24) of patients had no opinion on this subject. A total of 71% (17/24) of patients reported improved patient empowerment and a feeling of control over their illness, with the use of *the Box*, but 21% (5/24) of patients noticed no difference. A total of 8% (2/24) of patients did not answer this question. The reported issues were loss of Bluetooth connection with the devices (7/24, 29%), mainly the blood pressure monitor and signal noise when taking an ECG with the AliveCor Kardia (6/24, 25%). In addition, patients reported the use of all different mobile apps as time consuming (19/24, 79%).

#### Video Consultation

A total of 71% (17/24) of patients completed a video consultation. A total of 29% (7/24) of patients were unable to do so, with technical issues being the major reason (5/7, 71%). Some patients (3/24, 13%) experienced problems with either sound or video connection. Patients praised the fact that it was not necessary to come to the hospital, especially for people with mobility issues. One patient stated that the video consultation was effective but preferred a physical consultation, nonetheless.

#### Quality of Provided Support

Most patients (18/24, 75%) did not contact the helpdesk during the time they used *the Box*. Of the remaining 6 patients, 5 (21%) reported being happy with the service provided by the helpdesk, whereas 4% (1/24) of patients reported that issues were not resolved.

Most patients (18/24, 75%) stated that they preferred feedback on the measurements sent. A total of 17% (4/24) of patients recalled having noticed abnormal measurements, but only 4% (1/24) acted upon this. Of the other 3 patients, 2 (67%) expected to be contacted by *the Box* care team, and 1 (33%) patient did not want to bother the care providers.

#### Extra Items for Patients With a Low SES

A total of 90% (9/10) of patients with a low SES reported having issues with using the Health Mate and AliveCor Kardia apps because of the English instructions. A total of 50% (5/10) of patients stated that the terminology used in the manuals provided was too complicated to comprehend. A total of 30% (3/10) of patients stated that the use of more pictures would increase the functionality of the manuals and apps. Finally, 20% (2/10) of patients spontaneously mentioned that a reward system may be beneficial for their physical rehabilitation.

### Professional Experience

All NPs stated that when patients used *the Box*, fewer questions were asked, and the questions they had were more related to the illness, compared with non-Box patients. The NPs reported that the number of telephone consultations based on device readouts was low and did not interfere with their daily patient care. In addition, the possibility of looking up historical measurements and following a blood pressure trend were regarded as positive.

Video consultations provided a lower threshold to discuss topics such as sexuality and lifestyle without an increase in consultation time. However, professionals stated that difficulties with the internet connection at the patients’ homes interfered with the quality of the consultations done via the web. Equally, the necessity of a well-equipped and staffed technical support service was stressed as an important improvement for professionals.

## Discussion

### Principal Findings

#### Overview

*The Box* shows that a structural implementation of an mHealth initiative in daily outpatient clinic care is feasible in patients with cardiovascular disease (CVD). *The Box* has served 1140 patients within 4 years since its implementation, with a median participant’s age of 60.8 years. Most patients (839/1140, 73.59%) were male, which is most likely not explained by a higher mHealth engagement in men but rather by known sex differences in CVD leading to an overrepresentation of male patients [18-20]. *The Box* was well-received: patients described *the Box* as easy to use and reported an increased empowerment, providing them with more insight on their illness. NPs noticed this empowerment as well, as they described receiving fewer questions from patients and the questions being more on-topic compared with patients without a Box.

#### Trends in Box Distribution

As shown in Figure 3, it has been noticed that the number of patients receiving a Box is somewhat less during summer compared with the rest of the year. It is hypothesized that this is mainly because of fewer interventions (eg, AF ablations, cardiac surgery, and implantable cardioverter-defibrillator implantations) being carried out. After the outbreak of COVID-19 in the Netherlands in March 2020 [21], the initial rate at which boxes were handed out to patients with CVD has slowed down slightly because of a decline in the number of elective interventions. However, because of lockdown measures, physical outpatient clinic visits were cancelled and replaced by video consultations. To support this, more patients chose follow-up with *the Box* and concordantly, by video consultations instead of physical outpatient clinic visits without a Box. Moreover, a tailored Box was designed for patients with COVID-19. The timing of these adjustments correlates with the timing of the increase in the number of boxes. Thus, causality was assumed.

### Patient Experiences and Perspectives

#### Overview

*Box* users were overall satisfied with the care delivered via the mHealth care track. Distribution, installation, technical support, and ease of use were praised; however, internet and Bluetooth connection issues were frequently reported by users as troublesome. Identically, a feedback mechanism for sent measurements was noted by the majority of the interviewees as an important missing feature. These findings are in line with other studies that implemented eHealth care tracks in different health care domains [22-25]. eHealth satisfaction is generally high, as suggested by other sample studies [26,27]. Although evidence is scarce, internet connection and video quality issues are often mentioned to reduce satisfaction. These issues were also mentioned by the interviewees. This stresses the need for a strong digital infrastructure to support patients and professionals alike.

In the qualitative interviews, it was found that although satisfaction is high, patients find taking their own measurements time-consuming. This is possibly reflected in the relatively low median number of measurements taken, as shown in Table 2. However, there is no consensus on how to increase patient empowerment and participation; the concept of *gamification* could help to increase the number of measurements taken per patient [28,29]. This method has been proposed to improve patient behavior such as self-monitoring and could be investigated further for future improvements of *the Box* [30].

#### Feasibility of mHealth From the Patient’s Perspective

The findings of 4 years of clinical experience have indicated that eHealth is accepted by patients and that implementation is feasible. The results of our qualitative interviews indicate that patients become more active participants as they are asked to measure their own vitals daily. These findings were supported by the findings of an RCT in patients who had an MI [15]. These findings should be considered as hypothesis-generating and should be corroborated in future studies. A small minority of patients stopped using *the Box*. Of this group, the majority stated that taking daily measurements caused anxiety or distress rather than providing control over their disease. To some, it feels that they are continuously confronted with their illness, stigmatizing them. This effect has not yet been described in the literature; however, the negative effects of smartphone use on anxiety and stress levels have been described [31,32]. It is questionable how much an mHealth care track contributes to health care in patients who experience anxiety or another form of distress because of the service. Therefore, the extent and implications are being investigated as part of *the Box 2.0* [16].

### Comparison With the Literature

Often, mHealth studies use apps or other forms of guidance via participants’ mobile phones as an intervention rather than using mHealth devices such as a blood pressure monitor. For example, mHealth interventions in patients with chronic pain, diabetes mellitus, and mental health issues focus on improved information provision and strive for accessible ways to do so [33-35]. As these studies differ vastly from device studies, it was decided to only compare studies in which mHealth devices were used.

Lu et al [36] recently performed a meta-analysis of 11 RCTs to synthesize the effects of mHealth on blood pressure control, 9 of which used a self-monitoring blood pressure intervention. Participants were asked to measure their blood pressure up to 4 times a week and were followed up by telephone calls, SMS text messages, and emails. The mean participant age of these 9 studies varied from 57.0 to 67.4 years, with a median of 60.7 years. This is in line with the median age of 60.8 years of Box participants. One trial did not report the gender of the participants. Of the 3144 participants in the 8 remaining trials, 1752 (55.72%) were male. However, these studies were not carried out in patients with CVD but in patients with hypertension. The same is true for one of the largest mHealth trials, Assessment of Remote Heart Rhythm Sampling using the AliveCor heart monitor to screen for AF, which recruited 1001 participants >65 years via general practitioner records [37]. The mean age was 72.6 years (SD 5.4 years), and 46.55% (466/1001) of participants were males. A total of 50.05% (501/1001) of participants underwent an mHealth intervention, which consisted of acquiring a single lead ECG using an AliveCor Kardia twice weekly over 12 months. Although the mean age was high, most participants found it easy to use the device.

Few mHealth studies, focusing on those with CVD, can be compared with *the Box*. Oftentimes, studies only use 1 device, compared with up to 7 devices of *the Box* or a patch is studied for its diagnostic capabilities of rhythm disturbances [38-40]. One study carried out by McElroy et al [41] included 443 patients who underwent cardiac surgery. These patients underwent an intervention to reduce readmissions by offering improved education, including daily face-to-face sessions. Simultaneously, 27 patients who enrolled in a pilot project also received an mHealth intervention in the form of a so-called digital health kit, consisting of a blood pressure and heart rate monitor, a pulse oximeter, and a weight scale. However, it is unclear how these 27 patients were selected. The mean age of the 416 patients who received the improved education was 65.9 years (SD 14.1 years), with 65.8% (274/416) being male. The mean age of the 27 patients who received the mHealth intervention was 62.9 years (SD 9.8 years), with 85% (23/27) being male. However, the mHealth intervention did not significantly reduce the readmission rate; both patients and health care providers were satisfied with the intervention.

### Strengths and Limitations of the Box

*The Box* has strengths on both the patient and care provider level: it provides patients with more insight on their illness and engages them in their own care. Simultaneously, the NPs reported receiving fewer but more to-the-point questions from these patients. *The Box* has resulted in more outpatient clinic visits being replaced by webcam consultations. These consultations have been reported by both patients and health care professionals as accessible, more homelike, and productive. Therefore, *the Box* has become a major asset to the cardiology department of the LUMC.

**Figure 5:**
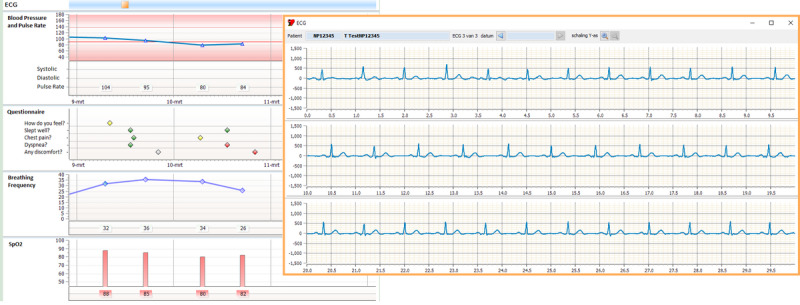
The user interface of the electronic medical record as of 2020, with incorporation of device and electrocardiogram data from the Withings Move electrocardiogram. ECG: electrocardiogram.

A limitation of *the Box* is the need for manual measurement checks. Although time is saved because of the replacement of outpatient contact moments with video consultations, NPs manually go through the list of measurements multiple times a week, creating a risk of data overload. Artificial intelligence may provide a solution to this problem. This is currently being investigated, together with engineers at the Delft University of Technology. However, as the project developed, EMR updates have provided NPs with a user interface for an easier overview of patients’ measurements, saving time compared with the start of the project. The most recent version of the EMR is shown in Figure 5.

Another limitation of our satisfaction analysis was the qualitative approach via interviews with a smaller sample. For a validated approach, satisfaction could have been measured more quantitatively with, for instance, the Telemedicine Usability Questionnaire [42].

Finally, it has to be noted that *the Box* was implemented in a tertiary care center, connected to a university. As *the Box* started with an RCT, part of the infrastructure was built for research purposes and funded via research grants. It is acknowledged that the setting might limit the external validity of the claim that *the Box* can be implemented in regular clinical care.

### Conclusions

In this study, the design and implementation of an mHealth care track in the outpatient clinic follow-up of patients with various CVDs was described. Data from these 4 years indicate that mHealth is feasible to incorporate in outpatient management and is generally well-accepted by patients and providers. Patient satisfaction is generally high, with patients praising its ease of use and educational capabilities. Providers commend on its ability to enhance patient engagement and medical literacy. Limitations include the need for manual measurement data checks and the risk of data overload. Moreover, the tertiary care setting in which *the Box* was introduced may limit the external validity of logistical and financial end points to other medical centers. More evidence is needed to show the effects of mHealth on clinical outcomes and cost-effectiveness.

## Abbreviations

AF: atrial fibrillation
CVD: cardiovascular disease
ECG: electrocardiogram
EMR: electronic medical record
GUCH: grown-ups with congenital heart
HF: heart failure
LUMC: Leiden University Medical Center
mHealth: mobile health
MI: myocardial infarction
NP: nurse practitioner
RCT: randomized controlled trial
SES: socioeconomic status
